# Dancing for Food in the Deep Sea: Bacterial Farming by a New Species of Yeti Crab

**DOI:** 10.1371/journal.pone.0026243

**Published:** 2011-11-30

**Authors:** Andrew R. Thurber, William J. Jones, Kareen Schnabel

**Affiliations:** 1 Integrative Oceanography Division, Scripps Institution of Oceanography, La Jolla, California, United States of America; 2 Environmental Genomics Core Facility, Environmental Health Sciences, University of South Carolina, Columbia, South Carolina, United States of America; 3 National Institute of Water and Atmospheric Research, Kilbirnie, Wellington, New Zealand; Biodiversity Insitute of Ontario - University of Guelph, Canada

## Abstract

Vent and seep animals harness chemosynthetic energy to thrive far from the sun's energy. While symbiont-derived energy fuels many taxa, vent crustaceans have remained an enigma; these shrimps, crabs, and barnacles possess a phylogenetically distinct group of chemosynthetic bacterial epibionts, yet the role of these bacteria has remained unclear. We test whether a new species of Yeti crab, which we describe as *Kiwa puravida* n. sp, farms the epibiotic bacteria that it grows on its chelipeds (claws), chelipeds that the crab waves in fluid escaping from a deep-sea methane seep. Lipid and isotope analyses provide evidence that epibiotic bacteria are the crab's main food source and *K. puravida* n. sp. has highly-modified setae (hairs) on its 3^rd^ maxilliped (a mouth appendage) which it uses to harvest these bacteria. The ε- and γ- proteobacteria that this methane-seep species farms are closely related to hydrothermal-vent decapod epibionts. We hypothesize that this species waves its arm in reducing fluid to increase the productivity of its epibionts by removing boundary layers which may otherwise limit carbon fixation. The discovery of this new species, only the second within a family described in 2005, stresses how much remains undiscovered on our continental margins.

## Introduction

**Figure 1:**
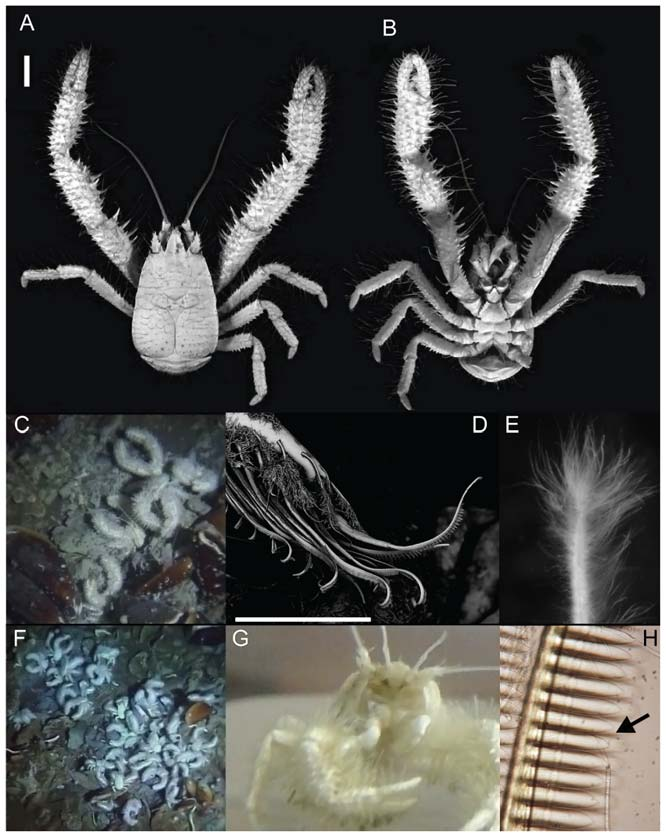
*Kiwa puravida* holotype, *in situ* images, and morphologic and behavioral adaptations to harvesting its bacteria. *Kiwa puravida* male holotype: (A) Dorsal view. (B) Ventral view (A and B Scale bar = 10 mm; Credit: Shane Ahyong, NIWA Wellington). (C) *in situ* next to Bathymodiolin mussels (D) Scanning Electron Micrograph of a detail of *K. puravida*'s 3^rd^ maxilliped and the comb-row setae which it uses to harvest its bacteria (scale bar = 150 µm credit; Shana Goffredi, Occidental College]. (E) Setae covered by bacteria from 3^rd^ pereopod (see Figure 4E for scale). (F) Dense aggregation *in situ*. (G) Shipboard photo of *K. puravida* using its 3^rd^ maxilliped to harvest its epibiotic bacteria. (H) Comb-row setae with bacteria filaments stuck among combs (indicated by arrow).

From ants that use symbiotic bacteria to protect the fungi that they farm [1] to polychaetes that “garden” bacteria [2], animals have developed a diversity of mechanisms to increase their symbionts' productivity and health. Few places provide as many examples of these symbioses as reducing systems, including hydrothermal vents and cold seeps, where shrimp, mussels, clams, and mouthless tubeworms are fueled by their chemoautotrophic bacterial symbionts [3]–[8]. As we try to understand how these systems function, novel species are constantly revealed. In 2005 a new family of crab was discovered at a hydrothermal vent. This crab had chelipeds (claws) covered in dense setae and epibiotic bacteria that lead to this species, *Kiwa hirsuta*, to be called the “Yeti crab” [9]. Only a single individual of *K. hirsuta* was collected leaving the ecology and role of its bacterial epibionts largely unknown [9], [10]. During June 2006, we discovered a second species of Yeti crab, which we formally describe here at *Kiwa puravida* n. sp, swinging its bacteria-laden chelipeds rhythmically at a Costa Rican methane seep (Figures 1A–C and 1F; Video S1). In this study, we describe how this new species farms its epibiotic bacteria in a unique form of symbiosis.

The symbiont- host interactions of epibiont bearing hydrothermal vent crustaceans have remained perplexing; these shrimp, barnacles, and crabs, share a distinct phylogeny of chemosynthetic epibionts [11] but if and how they harvest their symbionts has remained elusive (summarized in [8]). The best studied of this group is the vent shrimp *Rimicaris exoculata* that gains a large proportion of its energy from the ε- and γ-proteobacteria symbionts that grow inside its carapace and on its enlarged mouthparts [8], [12]–[15]. What remains unknown is how *R. exoculata* harvests the bacteria that grow on these structures. Competing hypotheses suggest that *R. exoculata* either uses its chelipeds to transfer its epibiotic bacteria to its mouth or that *R. exoculata* consumes its discarded bacteria-rich exoskeleton after molting [12]. This has led some to question the nutritional role of these symbionts [8]. Vent barnacles also appear to have morphological adaptation to harvest their symbionts [16], but no observations support this hypothesis. Only the vent crab *Shinkaia crosnieri,* has been observed to scrape off its epibiotic bacteria and transfer them to its mouth, providing a clear mechanism for symbiont harvesting [17]. Isotopic evidence further supported *S. crosnieri*'s consumption of its epibionts [18]. Yet the importance of *S. crosnieri*'s symbionts to its overall nutrition remains unknown [18]. The hydrothermal vent Yeti crab, *K. hirsuta,* may rely on its chemosynthetic symbionts for a food source, as suggested by the presence of enzymes necessary for carbon fixation within *K. hirsuta's* epibiotic bacteria, but it has not been observed to consume these bacteria [8], [10], [11].

A key aspect of farming, a form of mutualistic symbiosis, is the direct trophic transfer of energy from a symbiont to its host, a phenomenon that can be shown through biomarker analysis. Biomarkers, specifically carbon isotopic and fatty acid (FA) analyses, track a signature from an individual's diet into its tissues providing a time-integrated view of what that species eats [19], [20]. Chemosynthetic and photosynthetic derived biomass commonly differ in the ratio of carbon-13 to carbon-12 and the length, bonding and branching patterns of certain FAs in the producer's lipid membranes [21]–[23]. Consumers derive their isotopic and a portion of their FA composition from their diet [20], [24]. Through analysis of an individual's tissue, that individual's main food sources can often be identified. In certain cases, rare or unique FAs can be traced from a symbiont into their host, and provide a robust measure of direct consumption of that symbiont. An example of this is *R. exoculata*, as this shrimps' bacteria have relatively unique 16∶2(n−4) and 18∶2 (n−4) FAs that can be traced into the shrimp's tissue [25]. Perplexingly, these same (n−4) FAs were also present in *S. crosnieri*'s epibionts but they were not found in *S. crosnieri*'s tissue as would be expected if the crab's bacteria were its main food source [18]. Isotopic and FA analyses of *K. puravida* n. sp. may provide insight about its consumption of chemoautotrophic production and identify whether it is gaining energy from its symbionts or not.

Vents and seeps are both fueled by similar chemical reactions which has lead to cross-ecosystem comparisons since the first seep community was discovered [26]–[29]. Symbionts have been especially enlightening in demonstrating the similarity among these habitats [27], [29], [30]. An initial description of the epibionts found on the Costa Rican *Kiwa* species found provocative cross-ecosystem similarities among vent and seep crustacean epibionts based on a limited number of short (∼500 base pairs) 16S rRNA gene sequences [11]. Here we build upon that research through description of the host's phylogeny and add additional epibiont analysis. Our comparison of host and epibiont evolutionary histories will provide a better understanding of the biogeography of these disparate “islands” of chemoautotrophy in the deep-sea, as we test the hypothesis that *K. puravida* n. sp. farms its epibiotic bacteria.

## Materials and Methods

Specimens were collected with DSRV Alvin on RV Atlantis Cruises AT 15-5, AT 15-44, 15-59 June 16, 2006, February 22– March 23, 2009, and January 1–12, 2010, respectively, at Mound 12 off Costa Rica (8^o^ 55.8′N 84^o^18.8′W) at depths of 1000–1040 m. One specimen was collected at Mound 11 (8^o^ 55.2′N 84^o^18.2′W), although few individuals were observed there. Specimens were preserved in 8% buffered formalin or 95% ethanol. In two instances a pereopod was removed for isotopic, genetic, epibiont, and fatty acid analysis and frozen at −80°C.

Measurements of specimens are given in millimeters (mm) and indicate the postorbital carapace length unless otherwise indicated. Specimens are deposited at the Smithsonian (Holotype and Paratype 1) and University of Costa Rica (Paratype 2). Additional specimens are being deposited at NIWA Invertebrate Collection, Wellington, New Zealand, and the Scripps Institution of Oceanography Benthic Invertebrate Collection. Descriptions were prepared using DELTA (DEscriptive Language for Taxonomy [31]). Drawings were made using a WACOM Intuous3 and Intuous4 Graphics Tablets and Adobe Illustrator CS2–CS4.

Stable isotopic and fatty acid (FA) analyses were performed on two specimens that underwent molecular analysis and “bulk plankton” collected during cruise AT 15-5. The bulk plankton sample was collected less than 50 m above Mound 12 and sieved through a 63 µm sieve before being frozen at −80°C. Stable isotopic analysis was performed on muscle tissue of an additional 28 specimens, collected during cruises 15–44 and 15–59 at which time particulate organic carbon (POC) isotopic measurements were also made. POC isotopic analysis was performed on surface and bottom water at areas of active seepage along the Costa Rican margin. POC samples were collected by 9 CTD deployments and water was filtered through pre-combusted glass-fibre filters to estimate of the potential range of planktonic isotopic signature for this region. 0.2 mg of tissue, bulk plankton, or scraping of the GF/Fs were placed in tin boats, dried at 60°C overnight, acidified with 1% platinum chloride and analyzed on a Eurovector elemental analyzer interfaced with a continuous flow Micromass Isoprime isotope ratio mass spectrometer at Washington State University in the lab of Dr. Raymond Lee. Isotope ratios are expressed as δ^13^C in units of per mil (‰ - notation explained in [21]) using Pee Dee Belemnite as the standard. Fatty acids were extracted in a one step extraction-transesterification method [32]. Freeze-dried tissue was placed in 3 ml MeOH:HCl: CHCl_3_ (10∶1∶1 v/v/v) at 60°C for 60 min, cooled and 1 ml Milli-Q H_2_O added prior to extraction in hexane:chloroform, (4∶1 v/v), and dried over sodium sulfate. FAs were analyzed on a Thermo Finnegan Trace gas chromatograph/mass spectrometer and peak integration was performed using Xcaliber software. Percentage of total FAs are given for abundant FAs defined as those that composed more than 1.0% from any of the samples. As peak area measurements from mass spectra are not a function of concentration alone (spectra response varies with FA analyzed), these FA data are comparable within this study only.

Molecular analyses were performed on the two specimens collected during 2006. Genomic DNA was isolated from ethanol-preserved muscle (50 mg) and treated with the QiagenDNeasy isolation kit, according to manufacturer's instructions (Qiagen Inc., Valencia, CA). Polymerase chain reaction (PCR) conditions for amplification of the gene regions were as follows: 100 ng of template DNA, 5 µl 10X buffer (supplied by manufacturer), 5 µl MgCl_2_ (2.5 µM), 2 µl of each primer (10 µM final conc.), 2.5 units of Taq polymerase (Promega Inc., WI), 5 µl of a 2 mM stock solution of dNTPs, and sterile water to a final-volume of 25 µl. A ≈2000 basepair fragment of the 18 S rRNA gene was amplified with universal 18 S rDNA primers, 18e (5′-CTGGTTGATCCTGCCAGT-3′) and 18P (5′-TAATGATCCTTCCGCAGGTTCACCT-3′) [33]. PCR products were sequenced bidirectionally with an ABI 3100 DNA sequencer (Applied Biosystems Inc., Foster City, CA). GenBank 18 S rRNA sequences (Anomura: AF439381-AF439391, Z14062; *Upogebia affinis*: AF439392) were aligned with the yeti crab 18 S rRNA (DQ219316) using ClustalX^2^ followed by manual checking. Secondary structure of rRNA (i.e. stems and loops) was inferred using the program GeneBee [34]. Bayesian inference of phylogeny was performed using MrBayes v3.0B4 [35] with data partitions using RNA secondary structure. Six chains were run simultaneously for 1,100,000 generations and trees sampled every 1000 generations. The first 1000 trees were discarded as “burn in” and Bayesian posterior probabilities were estimated on the 95% majority rule consensus. The portion of the mitochondrial genome including Cytochrome Oxidase I (COI) and the intervening region to Cytochrome Oxidase II (COII) was amplified using previously published primers and conditions [9] to determine tRNA composition and order. 16 S rRNA from bacterial genomic DNA isolated from hairs on pereopods were amplified using 27F and 1492 primers. 16 S rRNA PCR products were cloned and sequenced using published protocols [10]. Separate Neighbor-Joining Trees were constructed to compare the ε-proteobacteria and γ-proteobacteria 16 S rRNA genes found on *K. puravida* n. sp. to sequences available in GENBANK. Sequence were selected for inclusion in the tree if they were identified as the most similar to sequences from reducing habitat decapoda and were greater than 1000 basepairs in length. The 16 s rRNA sequences were aligned using ClustalW within MegAlign (DNASTAR) followed by manual checking and trimming. 3000 iterations provided bootstrap confidence values and the trees were visualized using FigTree v1.3.1 with the ε- proteobacteria tree rooted by *Leucothrix mucro* (X87277) and γ-proteobacteria rooted with *Sulfurospirillum arcachonense* (Y11561).

## Results and Discussion

### Species Description

Family KIWAIDAE Macpherson, Jones & Segonzac, 2005.

Kiwaidae Macpherson, Jones and Segonzac, 2005: 712.

### Diagnosis

Body depressed, symmetrical. Carapace calcified, slightly convex, smooth. Rostrum well developed, triangular. Cervical grooves clearly distinct between gastric and anterior branchial regions and between anterior and posterior branchial regions; either side of mesogastric region with small sharply defined pit. Cardiac region small and depressed and separated from branchial regions by shallow grooves. Anterior branchial regions well delimited and separated by short median longitudinal groove; small W-shaped groove over this groove. Posterior branchial regions separated by median longitudinal groove. Intestinal region well circumscribed and separated from branchial regions by distinct grooves. Posterior half of pterygostomian flap with two longitudinal and subparallel carina. Abdominal segments smooth, not folded against thorax; telson folded beneath preceding abdominal somite, with a median transverse suture and a longitudinal suture in the posterior half of telson; uropods spatulate. Epistome unarmed. Mandibular cutting edge with chitinous teeth along incisor process. Sternal plate between third maxillipeds (sternite 3) well developed, strongly produced anteriorly; sternal plate between fifth pereopods (sternite 8) absent. Eyes strongly reduced to small soft tissue, not calcified, movable, without pigment, inserted near antennulae. Antennal peduncle 5-segmented, without antennal scale; flagellum of moderate length. Third maxillipeds with crista dentata in proximal half to third of ischium; epipods absent. Chelipeds (pereopod 1) strong, subequal, and greatly elongate; dense corneous spinules along distal portion of occlusal margin. Walking legs (pereopods 2–4) stout, with claw-like dactyli bearing dense corneous spinules along flexor margin. Fifth pereopod chelated, inserted below sternite 7, insertion not visible ventrally. Male first pleopod absent, pleopods 2–5 reduced, uniramous. Gills with four pairs of arthrobranchs (a pair each on P1–P4), 2 vestigial arthrobranchs on third maxilliped; pleurobranchs absent.

Genus *Kiwa* Macpherson, Jones & Segonzac, 2005

*Kiwa* Macpherson, Jones and Segonzac, 2005: 712

*Diagnosis.* — as for family

### Kiwa puravida n. sp

**Figure 2:**
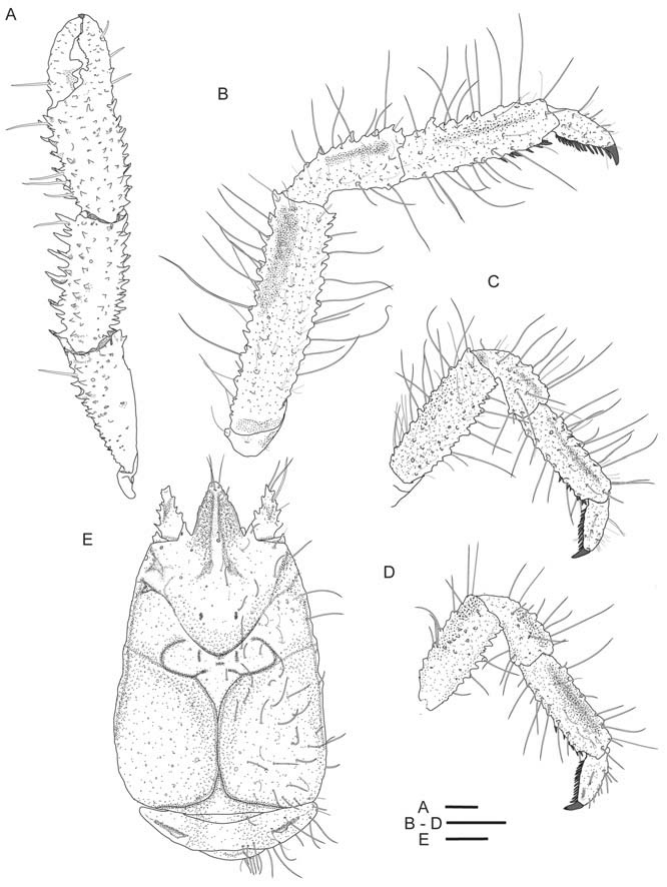
*Kiwa puravida* male holotype. (A) Pereopod 1 (cheliped). (B) Pereopod 2 (1st walking leg) (C) Pereopod 3. (D) Pereopod 4. (E) Carapace and abdomen. Scale bars = 5 mm.

**Figure 3:**
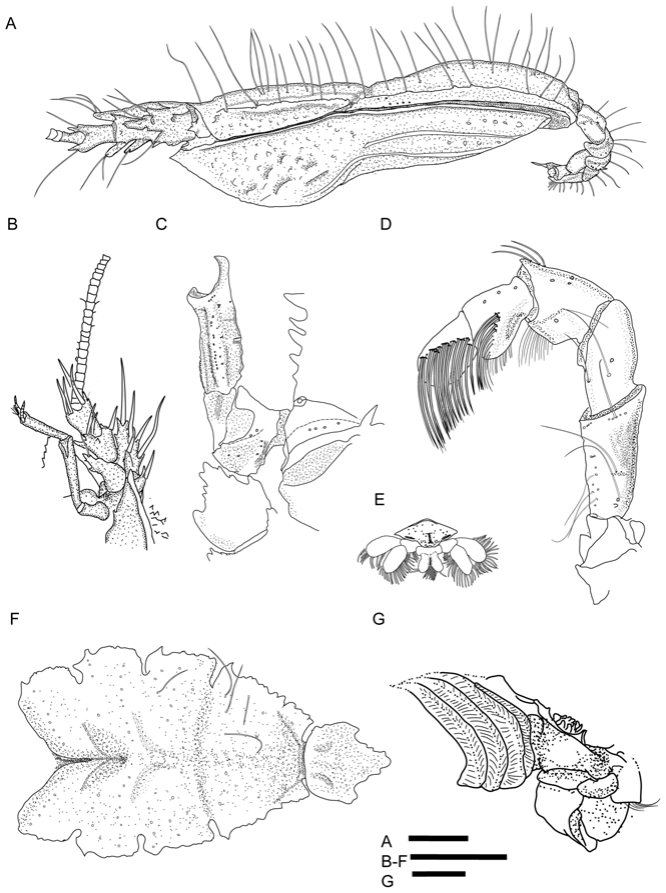
*Kiwa puravida* male holotype (A–F) , **male paratype (G).** (A) - Lateral view carapace and abdomen. (B) Antennule and antenna left and anterior part of ptergostomial flap. (C) Sternite 3, basis-ischium of third maxilliped, mesial view. (D) Third maxilliped. (E) Sixth segment of abdomen and telson. (F) Sternal plastron. (G) Male paratype gill arrangement. Scale bars = 5 mm.

**Figure 4:**
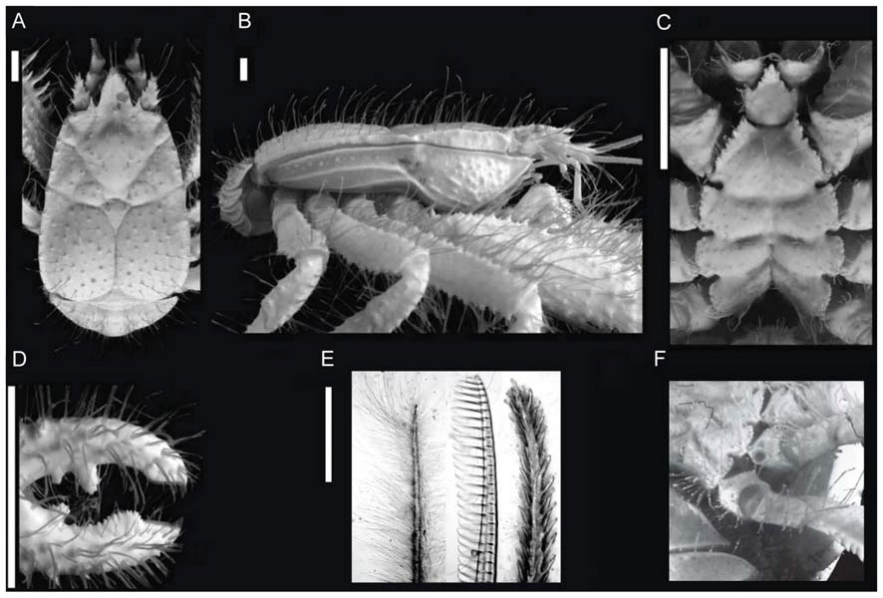
*Kiwa puravida* male holotype. (A) Dorsal carapace. (B) Lateral view. (C) Sternal plastron. (D) Finger and thumb on cheliped, (E) whip-like barbed setae (WBS, left), comb-row setae (middle), and stout barbed setae (SBS, right). (G) *K. puravida* female gonopore on third pereopod. Scale bars: A–D = 10 mm; E  = 500 µm. (A–D Credit: Shane Ahyong, NIWA Wellington).

Figures 1A and 1B; Figures 2, 3, 4.

### Material examined

#### Type specimens

HOLOTYPE: ♂ (30 mm), Costa Rica, 8^o^ 55.8′N 84^o^18.8′W, 1007 m, 15 May 2006, RV *Atlantis* and DSRV *Alvin*, Alvin Dive (AD) 4200, Scoop net (USNM 1160378). PARATYPE: ♂ (24.1 mm), same as holotype (USNM 1160379). PARATYPE: ♀ (21.5 mm), 997 m, 22 February 2009, RV *Atlantis* and DSRV *Alvin*, Biobox (MZUCR-2673-01).

### Other material examined

Juvenile (2.5 mm - SIO-BIC C11235), 997 m, 22 February 2009, RV *Atlantis* and DSRV *Alvin*. ♂ (9.5 mm - SIO-BIC C11237); ♀ (11.9 mm - SIO-BIC C11238); ♂ (14.1 mm - SIO-BIC C11239); ♂ (17.9 mm - SIO-BIC C11240); ♂ (30.2 mm - SIO-BIC C11241); ♀ (17.2 mm - SIO-BIC C11242); ♀ (4.7 mm SIO-BIC C11243), AD4511, March 5, 2009: ♂ (11.2 mm SIO-BIC C11244); ♀ (24.5 mm SIO-BIC C11245); ♀ (19.7 mm NIWA 70338); ♂ (20.9 mm NIWA 70338); ♀ (11.6 mm SIO-BIC C11248); Juvenile (3.9 mm SIO-BIC C11249). AD 4502, Feb. 23, 2009: ♂ (4.2 mm SIO-BIC C11250).

#### Etymology

Puravida, is a conjunction of the Spanish words “pura” and “vida” meaning pure life and is a common saying within Costa Rica, in whose waters these specimens were collected in. The genus is feminine as is the species name.

### Description

#### Carapace

1.3 times as long as broad (including rostrum) (1.5 times without rostrum). Dorsal surface unarmed and sparsely setose with stiff, barbed setae (SBS, Figure 4E, right image). Hepatic and epigastric regions depressed; distinct pit on either side in posterior gastric region. Region between the anterior and posterior portion of the cervical groove with distinct ∞-shaped groove. Frontal margin oblique, relatively straight, with large tooth near rostrum. Anterolateral margin rounded, lateral margin slightly divergent posteriorly (widest at distal quarter); slightly irregular with small granule at anterior margin of posterior branchial region, immediately posterior to deep groove. Posterior margin unarmed. Rostrum broadly triangular, horizontal, 0.2 times the length of remaining carapace; dorsal surface dorsally convex on either side of median ridge, smooth and sparsely furnished with SBS (dense cluster at apex); lateral margins slightly convex, irregular with granules but straight. Pterygostomian flap lateral surface granulate, with additional short striae in median portion and with two separate longitudinal carinae under posterior branchial region; anterior margin produced into a spine.

#### Sternum

sternal plastron 1.6 times as wide as long (at mid-length), convex lateral margins; surface smooth, sparsely furnished with fine whip-like barbed setae (WBS, Figure 4E left image), lateral margins serrated. Sternite 3, with strong spine at lateral midlength, anterior portion forming a roughly equilateral triangle, with large granules along lateral margins. Sternite 4 2.3 times as wide as sternite 3, anterior margin shallowly concave, anterior midline grooved. Anterolateral margin produced to tooth not overreaching sternite 3 and with large granules laterally. Sternite 6 widest. Posterior margin of sternite 7 deeply concave and deep median emargination.

#### Abdomen

tergites sparsely setose with SBS. Pleura each with anterior transverse carina and medially depressed; pleural margins of segments 2–6 strongly tapering and with concave anterior margins. Telson 1.1 times as broad as long; distal portions 2.6 times length of proximal portion, distally distinctly bi-lobed with median notch (0.4 times the length of distal portion).

#### Antennal peduncle

article 1 distomesial margin unarmed, distolateral margin produced to 3 small distal spines; article 2 with strong lateral projection reaching end of article 4, strongly toothed and with additional prominent ventral spine; article 3 laterally and distally toothed, nearly reaches distal end of segment 4. Penultimate article with 3 distal spines (2 mesial and 1 lateral); ultimate article armed with 1 dorsal and 1 ventral spine. Sparsely furnished with thick SBS.

#### Maxilliped 3

surface smooth and unarmed except for small granules at bases of plumose setae; coxa with fine serration along distal border, fully calcified (not corneous); ischium with 20 teeth on proximal third of mesial ridge. Two types of setae present including comb-row setae (CRS, Figure 1D and1H; Figure 4E center image) on ventral and distal carpus, propodus and dactylus and WBS on dorsal portion of all segments.

#### Pereopod 1 (cheliped)

strongly spinose, 2.6 times as long as carapace (excluding rostrum) (2.1 times including rostrum). Ischium with 2 dorsal distal spines, serrated along mesial margin. Merus with scattered strong spines, most prominent along mesial margin, with 6 distal spines. Carpus with multiple longitudinal rows of spines, with 6 distal spines, length of carpus slightly longer than palm. Propodus with palm 1.8 times as long as high, with distinct rows of spines. Length of dactylus 0.7 times as long as propodus, proximal tooth on lateral margin; occlusal margin distally hollowed, opposable margins strongly gaping proximally with prominent median tooth; fingers each distally with strong triangular corneous tooth. Ventral portion of ischium and merus densely furnished with WBS setae, remaining surfaces uniformly covered with SBS.

#### Pereopods 2–4

ambulatory legs similar. Surface covered with tubercular processes on meri, carpi and propodi. Merus dorsal margin with spines and large granules; with 8 spines on dorsal crest on P2 (including distal spine), ventral margin with row of tubercular processes, 1.4–1.0 times as long as propodus (for P2 and P4, respectively). Carpus, dorsal margin serrated with spines and tubercular processes. Propodus 2.1–1.9 times as long as dactylus (from P2–P4), extensor margin spinose, flexor margin with 6–7 corneous spines along distal third of margin, distal-most paired. Dactylus straight; flexor margin with 13, 12–15 inclined and slender corneous spines along entire length (P2, P3–P4, excluding distal spine). Dense fields of plumose WBS distributed along ventral portions of ishia and meri.

#### Gill structure

phyllobranchiate gills; 2 arthrobranchs each on the cheliped and the walking legs (P2–P4); pair of vestigial, lamellar gills on third maxilliped, none on P5; pleurobranchs are absent.

### Remarks

Neither of the original two specimens were complete, the male holotype specimen is lacking the posterior left two walking legs (P3 and P4), the male paratype specimen is lacking the right cheliped and the two posterior right walking legs. Sixteen additional specimens were collected during 2009 which included 6 females between carapace length (including rostrum) 4.9 and 24.5 mm and 7 male specimens between 7.4 and 38.6 mm. A single specimen, 4.6 mm, had potentially developing gonopores, indicating that it was female and this specimen and those smaller were classified as juveniles. Female gonopores were clearly visible making identification of sex possible (Figure 4F). A single gravid female was collected that had 88 eggs that were approximately 1 mm in diameter.

The overall morphology and morphometrics of the paratypes and other specimens examined agree with the holotype, except for a variance in the shape of the rostrum. The rostrum of one of the paratypes is not evenly triangular but more leaf-shaped along the left margin.

*Kiwa puravida* n. sp. is similar to *K. hirsuta* [9] but can be clearly distinguished by the following characters:

frontal margin with prominent tooth at base of rostrum in *K. puravida* n. sp. (*K. hirsuta* only has a small tooth; Figure 2E and 4A).
pteryogostomian flap covered with large granules and short striae in addition to longitudinal carinae, anteriorly produced to spine and with short, scattered setae (*K. hirsuta* only has a few scattered granules in the anterior portion, anteriorly rounded and without setae; Figure 3A and 4B).
the proportions of the anterior and posterior portions of the telson differ with *K. puravida* n. sp. having a distinctly larger posterior portion (1.6 to 2.4 times a long as anterior portion) compared to *K. hirsuta* (0.8). Additionally, the distal cleft of the telson is distinctly deeper in the new species with the distal portion of the telson in *K. hirsuta* being only shallowly emarginated (Figure 3F and 4C). The extent of the cleft was reduced in a specimen whose carapace was 4.7 mm and should not be used as a distinguishing feature in small specimens.
anterior portion of sternite 3 in the shape of a wider triangle with length-to-width ratio 0.7 (*K. hirsuta* sternite 3 anterior portion is acute with approximate length-to-width ratio of 1.4; Figure 3F and 4C).
anterior portion of sternite 4 is shallowly concave (*K. hirsuta* anterior margin of sternite 4 is deeply concave; Figure 3F and 4C).
lateral margins of sternal plastron are distinctly serrated (smooth in *K. hirsuta*; Figure 3F and 4C).
all articles of the antennal peduncle are distinctly more spiny and with the prominent lateral process on article 2, reaching the distal margin of article 4 (instead of only reaching to midlength in *K. hirsuta*; Figure 3C).
the coxa of the third maxilliped is distally not strongly produced, only slightly serrate and without corneous spines (distal border strongly produced and denticulate with each tooth with corneous margin in *K. hirsuta;* Figure 3D).
each tip of the cheliped fingers bear a single corneous spine only (Figure 4D) while in *Kiwa hirsuta* bears two corneous tips on the fixed finger.
propodi of walking legs with 4–8 movable corneous spines along distal half portion of flexor margin (*K. hirsuta* has 11–16 spines along nearly the entire margin Figure 2B–2D).

The various types of setae that cover the surfaces of the body and appendages in both species remain intriguing. Macpherson et al. [9] describe the setation as dense long plumose setae (similar to WBS) mainly on sternum and ventral surface of pereopods and rigid chitinous seta with barbules (analogous to our SBS) inserted in pairs mainly on the merus of the cheliped. Unlike the rigid chitinous setae of *K. hirsuta*, the SBS of *K. puravida* n. sp. were covered with bacteria, even though the density of bacteria was much reduced compared to the WBS. The barbules on WBS were not easily visible except under 200x power magnification. The CRS, which were not reported on *K. hirsuta,* were limited to the carpus, propodus and dactylus of the third maxilliped (Figure 1D and 4E). The second maxilliped of *K. puravida* n. sp. had CRS present but with much reduced combs.

The diagnosis of the family Kiwaidae and genus *Kiwa* is adjusted; the presence of the row of corneous spines on the coxa of the third maxilliped is excluded as this character varies between the two species now known in genus *Kiwa.* However, the presence of a dense row of corneous spinules along distal margins of the cheliped fingers is added. Furthermore, the gill structure appears to be unique in this family compared to other anomurans, specifically involving the presence of only four pairs of arthrobranchs with gills absent from the third maxilliped. The closely related Chirostylidae have an additional pair of arthrobranchs on the third maxilliped. Albuneids, hippids and aeglids are comparably more similar in having a pair of arthrobranchs on P1–P4 and a single arthrobranch on the maxilliped [36].

Additionally, the two *Kiwa* species differ in their habitat. The new species was collected from a methane seep at 1000-m depth while *K. hirsuta* was observed and collected at greater depth (exceeding 2200 m) at a hydrothermal vent. The collection locations of *K. hirsuta* and *K. puravida* n. sp. are 6,500 km apart.

### Nomenclatural Acts

The electronic version of this document does not represent a published work according to the International Code of Zoological Nomenclature (ICZN), and hence the nomenclatural acts contained in the electronic version are not available under that Code from the electronic edition. Therefore, a separate edition of this document was produced by a method that assures numerous identical and durable copies, and those copies were simultaneously obtainable (from the publication date noted on the first page of this article) for the purpose of providing a public and permanent scientific record, in accordance with Article 8.1 of the Code. The separate print-only edition is available on request from PLoS by sending a request to PLoS ONE, 1160 Battery Street, Suite 100, San Francisco, CA 94111, USA along with a check for $10 (to cover printing and postage) payable to "Public Library of Science".

In addition, this published work and the nomenclatural acts it contains have been registered in ZooBank, the proposed online registration system for the ICZN. The ZooBank LSIDs (Life Science Identifiers) can be resolved and the associated information viewed through any standard web browser by appending the LSID to the prefix "http://zoobank.org/". The LSID for this publication is:

urn:lsid:zoobank.org:pub:B59E7704-9AD0-4D45-A5E2-6EC8C00E9508

The LSID for *Kiwa puravida* n. sp. is:

urn:lsid:zoobank.org:act:36679362-65B9-4CE9-840A-9D1AF4A9DFD9

### Phylogenetic affinities

**Figure 5:**
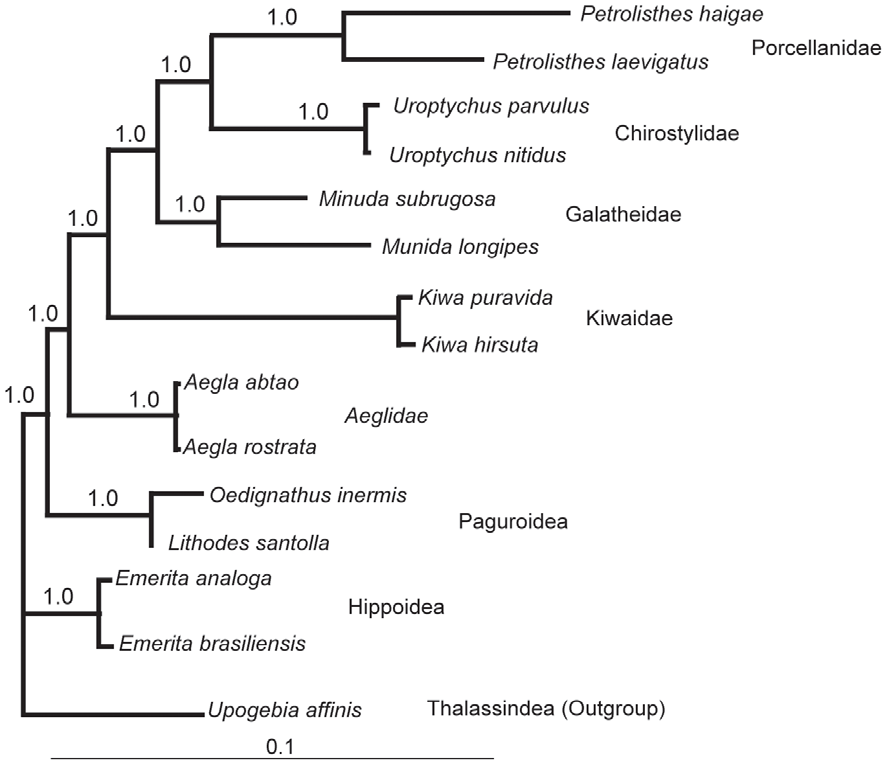
Bayesian phylogenetic tree based of 18 S rRNA, rooted using *Upogebia affinis*. Scale bar equals percent sequence divergence.

Both morphology and molecular data support *K. puravida* n. sp. as a new species of Kiwaidae. The type species of the Kiwaidae, *K. hirsuta* that was collected from 2228 m at an eastern Pacific hydrothermal vent, is closely related to but genetically distinct from the methane-seep species described here, having a COI nucleotide similarity of 86% and 18 S rRNA gene sequence similarity of 98% (GenBank Accession Number JN383822 for COI and JN367460 for18 S rRNA; Figure 5). Furthermore, both *Kiwa* species have four tRNAs between COI and COII in the mitochondrial genome (Leu(CUN), Leu(UUR), Ala, Gly); this appears to be a unique feature to Kiwaidae (see [37]–[39]). *Shinkaia crosnieri* and *K. puravida* are distinct both morphologically and genetically as well, having only 77% COI nucleotide similarity, which conforms with the family-level differences between these two taxa [40]. Although COI is a poor determinant of higher level relationships, no 18 S rRNA gene sequence data are currently available for *S. crosnieri* leaving COI data as the only available comparison between *Kiwa* (Kiwaidae) and *Shinkaia* (Munidopsidae) families.

A total of 26 full-length 16 S rRNA bacterial gene sequences were collected from a single individual of *K. puravida* n. sp. that included 17 ε-proteobacteria, four δ-proteobacteria, two γ-proteobacteria, and three bacteroides phylotypes (Table 1; GenBank accession JN255989-JN256014). The γ-proteobacteria recovered from *K. puravida* n. sp. were similar to the other epibionts collected from reducing-habitat decapods, including *K. hirsuta, S. crosnieri* and *R. exoculata*, being 97% and 98% similar among these four taxa. The sequenced ε-proteobacteria fell within the Marine Group 1, Thiovulgaceae, and all of the ε-proteobacteria were between 95–98% similar to epibionts of *K. hirsuta* and *S. crosnieri* and 95–96% similar to epibionts of *R. exoculata* (Table 1). The δ-proteobacteria and bacteroides were most similar to environmental samples collected from cold seep, hydrothermal vent, and cave systems with complex sulfur cycling.

**Table 1:** Phylogenetic affinity of bacterial epibionts on *Kiwa puravida*.

		Most similar sequence			*Kiwa hirsuta*		*Shinkaia crosnieri*		*Rimicaris exoculata*	
Group	Accession #	Source	% Sim	Accession #	% Sim	Accession #	% Sim	Accession #	% Sim	Accession #
ε-Proteobacteria	JN255994	*S. crosnieri* Epibiont	96%	EU107475	96%	EU265786	96%	EU107475	96%	FM203406
	JN256000	*S. crosnieri* Epibiont	96%	EU107475	96%	EU265787	96%	EU107475	96%	FM203395
	JN255996	*S. crosnieri* Epibiont	97%	EU107475	96%	EU265786	97%	EU107475	96%	FM203406
	JN255989	*S. crosnieri* Epibiont	97%	EU107475	96%	EU265787	97%	AB440170	96%	FM203395
	JN255993	*S. crosnieri* Epibiont	96%	EU107475	95%	EU265786	96%	EU107475	96%	FM203406
	JN256003	Peltospiridae Epibiont	96%	AY531601	95%	EU265793	96%	AB476188	95%	FN658695
	JN256002	*S. crosnieri* Epibiont	96%	EU107475	96%	EU265785	96%	EU107475	96%	FM203396
	JN255991	*S. crosnieri* Epibiont	97%	EU107475	96%	EU265787	97%	EU107475	96%	FM203395
	JN255998	*S. crosnieri* Epibiont	96%	EU107475	96%	EU265786	96%	EU107475	96%	FM203406
	JN255992	*S. crosnieri* Epibiont	96%	EU107475	95%	EU265785	96%	EU107475	95%	FM203395
	JN255997	*S. crosnieri* Epibiont	96%	EU107475	95%	EU265786	96%	EU107475	96%	FM203406
	JN255999	*S. crosnieri* Epibiont	97%	EU107475	96%	EU265786	97%	EU107475	95%	FM203395
	JN255995	*S. crosnieri* Epibiont	96%	EU107475	95%	EU265787	96%	EU107475	96%	FM203406
	JN256001	*K. hirsuta* Epibiont	96%	EU265787	96%	EU265787	96%	EU107475	95%	FM203395
	JN255990	*S. crosnieri* Epibiont	97%	EU107475	96%	EU265787	97%	AB440170	96%	FM203395
	JN256005	Hydrothermal Bacterial Mat	96%	AY075127	95%	EU265787	96%	AB440170	95%	FM203377
	JN256004	*K. hirsuta* Epibiont	98%	EU265786	98%	EU265786	98%	EU107475	96%	FN393028
δ-Proteobacteria	JN256009	HR Methane Seep Sediment	98%	AJ535238	95%	EU265788	84%	AB476257	96%	FN658700
	JN256010	HR Methane Seep Sediment	99%	AJ535238	95%	EU265788	81%	AB476262	96%	FN658700
	JN256011	Peltospiridae Epibiont	94%	AY531586	89%	EU265788	81%	AB476257	89%	FN658700
	JN256008	Peltospiridae Epibiont	93%	AY355303	89%	EU265788	82%	AB476257	91%	AM412517
Bacteroides	JN256012	Methane Seep Sediment	98%	FN658702	80%	EU265794	87%	AB476251	98%	FN658702
	JN256013	Haakon Mosby Sediment	98%	AJ704707	92%	EU265797	87%	AB476194	81%	FN658702
	JN256014	Peltospiridae Epibiont	97%	AY531558	80%	EU265794	87%	AB476273	97%	FN658701
γ- Proteobacteria	JN256006	*K. hirsuta* Epibiont	98%	EU265784	98%	EU265784	98%	AB476177	98%	FM203402
	JN256007	*K. hirsuta* Epibiont	97%	EU265791	97%	EU265791	97%	AB476177	97%	FM203375

Relationship between *Kiwa puravida* epibiont fauna and sequences available in GenBank as of 9/12/2011 using 16 S rRNA molecular data. All bacterial epibiont sequences were observed at least twice during sequencing.

**Figure 6:**
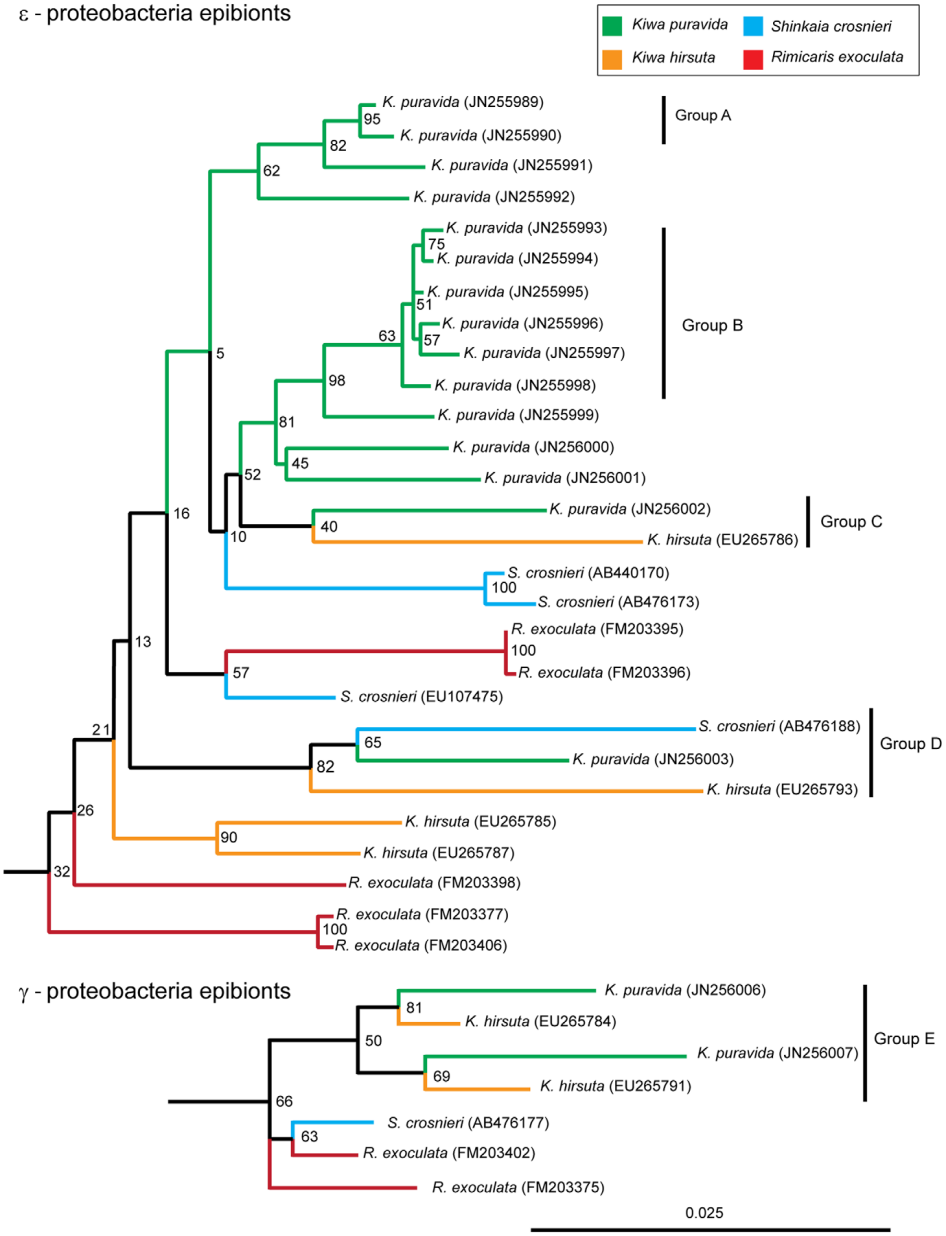
Neighbor-Joining phylogenetic tree based of 16 S rRNA of ε- and γ-proteobacteria of epibionts found on *Kiwa puravida* n. sp. Tree's were rooted using *Leucothrix mucor* (X87277) and *Sulfurospirillum arcachonense* (Y11561) for ε- and γ- proteobacteria, respectively, and the roots are not shown. Sequence from Table 1 were excluded from these trees if they were less than 1000 base pairs. Bootstrap confidence, based on 3000 iterations, are indicated in grey. Scale bar equals number of substitutions per nucleotide.

A phylogenetic analysis of ε- and γ-proteobacteria found that there was poor support for the relationships displayed within the trees except in six host specific clades (Figure 6). Two clades of ε- proteobacteria were unique to *K. puravida* n. sp. (Groups A and B) and were separate from the remaining branches in 82% and 98%, respectively, of the possible trees. Two of the *K. puravida* n. sp. ε-proteobacteria phylotypes were more similar to phylotypes from other species than those collected on the same host (Figure 6; Groups C and D). Intriguingly, within the γ-proteobacteria, a *Kiwa* clade including both species' epibionts was formed (Group E), separating these phylotypes from the remaining similar sequences. Bootstrap confidence for this clade was low at 66% yet in 81% of the possible trees a hydrothermal phylotype (EU265784) and the methane-seep phylotype (JN256007) were found adjacent to each other. The higher level relationship among the γ-proteobacteria epibionts from each of the other taxa were not well defined. The poor bootstrap support for the location of the basal branches of both trees was as would be expected for sequences that were similar to each other and thus small sequence differences would cause large impacts to the location of each branch of the tree.

Both the similarity analysis (i.e. Table 1) and the phylogenetic analysis supported the hypothesis that there is an epibiotic fauna that specializes on inhabiting both vent and seep decapods. Thus our full length 16 s rRNA gene sequences corroborate the findings of Goffredi [11] who inserted comparably shorter reads into a full length constructed tree. The presence of closely related γ- and ε- proteobacteria on diverse hydrothermal-vent hosts has been suggested to be evidence of multiple horizontal transmission events by vent-species epibionts [8]. Furthermore, Peterson et al. [8] hypothesized that, as it is unlikely that the host species co-occur at any one site, these similarities indicate that there are free living bacterial stages of these symbionts that disperse among hydrothermal-vent sites. Since we have now found both lineages at a methane seep, specifically on *K. puravida* n. sp, we can include methane seeps as likely stepping stones among hydrothermal vent locations. Furthermore, it appears that the one *K. puravida* n. sp. individual studied had two distinct clades of ε- proteobacteria and potentially two clades of γ- proteobacteria co-occurring on its setae. The presence of both bacterial phyla with high similarity to epibionts at hydrothermal vents suggests that *K. puravida* n. sp. may possess a duel symbiosis similar to that found on *R. exoculata* [8]. As our bacterial phylogenetic study results deals with sequences from a single individual and has not identified the distribution of the bacterial phylotypes even within that species (such as through fluorescent in situ hybridization analysis) the results are largely preliminary. Yet it clearly shows the importance of including methane-seep fauna when trying to understand the biogeography of hydrothermal-vent habitats, highlighting the importance of studies such as Goffredi [11].

### Nutrition and Farming

During submersible dives with the DSRV Alvin off of Costa Rica, *K. puravida* n. sp. were observed to have a patchy distribution on the tops and within crevices of carbonate outcroppings, on *Lamellibrachia* cf. *barhami* colonies, amongst bathymodiolin mussels, and in pits within carbonate rocks with alvinocarid shrimp (Figure 1C and 1F). The presence of these other symbiont-bearing species indicates that these areas have active methane-seep fluid release. This new *Kiwa* species was not observed scavenging food, a strategy previously suggested for its congener, *K. hirsuta* [9]. In addition, *K. puravida* n. sp. were often seen using their chelipeds to force shrimp away that got close to the crab; the crab made no obvious attempt to capture these shrimp. However, individuals were commonly observed slowly waving their chelipeds (pereopod 1) back and forth in these areas of active seepage (Video S1).

**Figure 7:**
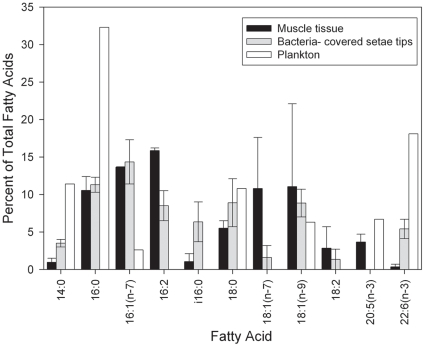
Fatty acid composition of muscle tissue and bacteria-laden setae in relation to plankton. Mean fatty acid composition of abundant (>1‰ in any sample) fatty acids of muscle and bacteria-laden setae from 3rd or 4th pereopod of *Kiwa puravida* with the fatty acid composition of plankton from the overlying water column. Error bars = range. N = 2.

Both isotopic and FA biomarker approaches provided multiple lines of evidence to indicate that *K. puravida* n. sp. used its epibiotic bacteria as a main food source. The carbon isotopic composition of this species (δ^13^C_muscle_ = −20.1 to −44.2 ‰, n = 30) was significantly lighter than phytoplanktonic production in this community (δ^13^C_plankton_ = −16 to −21 ‰, n = 18: T-test t = −8.9, df = 42.8, p<0.001), clearly indicating a chemoautotrophic food source for *K. puravida* n. sp. The light and wide ranging isotopic values of this species indicate that sulfide- and potentially methane- oxidation fuel this chemoautotrophic symbiosis [21]. *Kiwa puravida* n. sp. 's muscle FA profile was also divergent from the planktonic FA composition and mirrored that of its bacteria-laden setae (Figure 7). The FA_plankton_ composition included an abundance of polyunsaturated fatty acids (PUFAs) with 18% of the FA composition made up by 22∶6(n−3) and 7% from 20∶5(n−3); both diagnostic for photosynthetic production ([20] but see [41]). In contrast, 20∶5(n−3) was not common in the *K. puravida* n. sp. tissue, composing only 3 and 5% of the FAs measured in the two specimens tested and this FA was never observed in either of the specimens' setae sampled. 22∶6(n−3) was present between 4 and 1% in the tissue and was present in one of the specimens' setae sampled, comprising 6% of that species total FAs. 16∶2 and 18∶2 FAs, which are abundant in *R. exoculata* symbionts [25], were present on *K. puravida* n. sp. 's bacteria-setae and tissue sample with the majority being within the tissues; the sum of the two diene FAs comprised 33% of both specimens' tissue samples. 16∶2 biomarkers have also been found in limited abundance in mussel tissues [42] and diatoms [43] yet were not found in a species of bathymodiolin mussel from Costa Rica (Thurber, Pers. Obs.) or the phytoplankton sample collected. The relative input of free living microbes to *K. puravida* n. sp. from carbonate rock was not tested, a food source thought to augment the diet of *R. exoculata* [15], [44]. We did not collect the rock that *Kiwa* were discovered on, although active carbonate rock from the same seep did not have 16∶2 FAs (Thurber, Pers. Obs.). The isotopic composition of this species does not eliminate the possibility that *K. puravida* n. sp. may be a scavenger or consume symbiont-bearing fauna, but we did not observe them grazing upon other fauna and the unique 16∶2 FA within their tissues further makes this lifestyle less probable. The monounsaturated fatty acid, 16∶1(n−7), a common constituent of sulfide-oxidizing bacteria [45], was always more than four times as abundant in tissue and spines of *K. puravida* n. sp. compared to the plankton sample. The presence of 16∶2 FA, an abundance of 16∶1(n−7), the similarity of bacteria-laden setae with muscle tissue, and the isotopic composition indicative of chemosynthetic nutrition, support the hypothesis that *K. puravida* n. sp. 's main food source is the epibiotic bacteria growing on its setae.

In addition to this biomarker evidence, *K. puravida* n. sp. possessed both morphological and behavioral adaptations to harvest its epibionts. *Kiwa puravida* n. sp. had specialized CRS setae on its 3^rd^ maxilliped (a mouth appendage; Figure 1D and present in all 3 individuals inspected via light or scanning electron microscopy). The crab uses its CRS to scrape bacteria off of the WBS which adorn its chelipeds, sternum, and pereopods and then transfers the harvested bacteria into its mouth (Figure 1G, Video S2). Dissection of a single individual revealed that the bacterial filaments were commonly stuck within these CRS, indicating that the crab was effective at removing the filaments from its setae (Figure 1H). Bacterial filaments were found in the same individual of *K. puravida* n. sp. 's cardiac stomach supporting the observation that these bacteria are transferred into its mouth, an observation similar to what has been recorded in *R. exoculata* [46]. However, a gut-specific microbiota are known to occur in *R. exoculata* that are similar in appearance to those that grow upon the shrimp epibiotically [47]. Thus a molecular identification is necessary to confirm that the bacteria found in the cardiac stomach of *K. puravida* n. sp. is the same bacteria that grow on the WBS. Yet the bacteria observed in the gut did have the same morphologic structure as those found growing epibiotically on this new crab species.

In addition to bacteria, detritus was observed both attached to the bacteria-laden setae (in all specimens observed) and was present within the *Kiwa* mouth of the individual that was dissected. As potential photosynthetic fatty acid biomarkers were observed, albeit minimally, both within the tissue and the setae, it seems likely that this species may periodically augment its diet through sweeping up detritus, the source of this photosynthetically-derived particulate organic matter. Yet this did not appear to be the main food source for *K. puravida* n. sp. as shown by the stable-isotopic composition of this species.

For a species to farm bacteria it must facilitate the growth of its epibionts. In a minimalist sense, *K. puravida* n. sp. does this by providing an attachment substrate for its bacteria, yet through its continual cheliped movement, *K. puravida* n. sp. likely facilitates increased epibiont productivity as well. Chemoautotrophic symbionts require access to oxygen from the water column and reduced compounds, i.e. sulfide or methane, from the seep. During periods of carbon fixation, a boundary layer depleted in one or more of these solutes likely develops, which limits epibiont productivity. This is analogous to reef-building corals whose photosynthetic symbionts become carbon limited during periods of high productivity [48]. In coral symbioses, carbon limitation becomes ameliorated at increased current speeds or mixing of the water column which replenishes these boundary layers [49], [50]. We hypothesize that *K. puravida* n. sp. waves its chelipeds to shear off boundary layers formed by their epibionts productivity, increasing both the epibionts and, in turn, their own access to food. As in corals, boundary layers are greatest in areas of reduced flow, such as pits and depressions [51], which is a habitat where *K. puravida* n. sp. commonly occurs, making this behavior even more adaptive. Thus the cheliped waving motion may increase its epibionts chemoautotrophic productivity and yield.

### Ecology and Behavior

In addition to its bacteria harvesting, this species demonstrated intriguing intra-species interactions (Video S3). An individual that appeared to have recently molted due to its minimal bacterial covering, began grappling with a larger specimen that it approached. This ended in a dominance display where the challenged individual forced the challenging individual off the carbonate outcropping while both individuals had their chelipeds spread apart. As decapods commonly reproduce after molting, as has also been observed in the hydrothermal vent *S. crosnieri* [17], the individual that was forced off may have been inseminated during this display or this may have been a behavior demonstrating how this species competes for space in areas of active seepage.

A remaining question is: do *K. puravida* n. sp. setae act as mechanoreceptors allowing it to identify areas of active seepage? A similar role has been suggested for the setae of both *S. crosnieri* and *K. hirsuta* [52]. Through analysis of the setal structure of *S. crosnieri* and *K. hirsuta* and the mitten crabs, specifically *Eriocheir sinensis* and *E. japonica,* all of which are characterized as having dense setae, Ehrlich [52] concluded that it is unlikely that the setae of these species posses a mechanoreceptor ability. As *K. puravida* n. sp. has three distinct setae types, morphological analysis would be a useful approach to see if any of the distinct setae forms act as these mechanoreceptors in contrast to the previously studied highly-setose anomurans.

The nutritional role of epibionts that grow on hydrothermal vent decapods has remained perplexing. *Kiwa hirsuta* is known from only one specimen thus many analyses have not been performed to identify its food [9]. *Rimicaris exoculata* has not been observed harvesting its epibionts and doesn't possess the morphological structures to do so [9]. *Shinkaia crosnieri* posses the behavior and morphological adaptations to consume its epibiotic bacteria and stable isotopic evidence further supports that it consumes its epibiotic bacteria, but this species does not have a FA signature indicative of a diet based on the bacteria that grows upon it [18]. The multiple lines of evidence that we present here for *Kiwa puravida* n. sp. together demonstrate clearly that this new species of yeti crab farms the bacteria that grow upon it in a novel form of symbiosis.

## Supporting Information

Video S1 ***Kiwa puravida*** **at Mound 12, Costa Rica demonstrating the rhythmic waiving of its chelipeds.**
(MP4)

Video S2 ***Kiwa puravida*** **harvesting its symbionts with its 3rd maxilliped.** After scraping bacteria off of its pereopods and sternum *K. puravida*'s transfers the bacteria to its mouth with the aid of its 2nd maxillipeds.
(MP4)

Video S3 **Two individual of** ***K. puravida*** **performing either a courtship or competitive display.**
(MP4)

## References

[1] Currie CR, Scott JA, Summerbell RC, Malloch D (2001). Fungus-growing ants use antibiotic-production bacteria to control garden parasites.. Nature.

[2] Grossman S, Reichart W (1991). Impact of *Arenicola marina* on bacteria in intertidal sediments.. Mar Ecol Prog Ser.

[3] Felbeck H (1981). Chemoautotrophic potential of the hydrothermal vent tube worm, Riftia pachyptila Jones (Vestimentifera).. Science.

[4] Cavanaugh CM, Gardiner SL, Jones ML, Jannasch HW, Waterbury JB (1981). Prokaryotic cells in the hydrothermal vent tube worm *Riftia pachyptila*: Possible chemoautotrophic symbionts.. Science.

[5] Childress JJ, Fisher CR, Brooks JM, Kennicutt MC, Bidigare R (1986). A methanotrophic marine molluscan (Bivalvia, Mytilidae) symbiosis: mussels fueled by gas.. Science.

[6] Schmaljohann R, Faber E, Whiticar MJ, Dando PR (1990). Co-existence of methane- and sulpher-based endosymbioses between bacteria and invertebrates at a site in the Skegerrak.. Mar Ecol Prog Ser.

[7] Duperron S, Nadalig T, Caprais J-C, Sibuet M, Fiala-Médioni A (2005). Duel symbiosis in a *Bathymodiolus* sp. mussel from a methane seep on the Gabon Continental Margin (Southeast Atlantic): 16 S rRNA phylogeny and distribution of the symbionts in gills.. Appl Environ Microbiol.

[8] Petersen JM, Ramette A, Lott C, Cambon-Bonavita M-A, Zbinden M (2009). Dual symbiosis of the vent shrimp *Rimicaris exoculata* with filamentous gamma- and epsilonproteobacteria at four Mid-Atlantic Ridge hydrothermal vent fields.. Environ Microbiol.

[9] MacPherson E, Jones W, Segonzac M (2005). A new squat lobster family of Galatheoidea (Crustacea, Decapoda, Anomura) from the hydrothermal vents of the Pacific- Antarctic Ridge.. Zoosystema.

[10] Goffredi SK, Jones WJ, Erhlich H, Springer A, Vrijenhoek RC (2008). Epibiotic bacteria associated with the recently discovered Yeti crab, *Kiwa hirsuta.*. Environ Microbiol.

[11] Goffredi SK (2010). Indigenous ectosymbiotic bacteria associated with diverse hydrothermal vent invertebrates.. Environ Microbiol Rep.

[12] Gebruk AV, Pimenov NV, Savvichev AS (1993). Feeding specialization of bresiliid shrimps in the TAG site hydrothermal community.. Mar Ecol Prog Ser.

[13] Gebruk AV, Southward EC, Kennedy H, Southward AJ (2000). Food sources, behavior, and distribution of hydrothermal vent shrimps at the Mid-Atlantic Ridge.. J Mar Biol Ass U K.

[14] Segonzac M, Desaintlaurent M, Casanova B (1993). Enigma of trophic adaptation of the shrimp Alvinocarididae in hydrothermal areas along the Mid-Atlantic Ridge.. Cah Biol Mar.

[15] Polz MF, Robinson JJ, Cavanaugh CM, van Dover CL (1998). Trophic ecology of massive shrimp aggregations at a Mid-Atlantic Ridge hydrothermal vent site.. Limnol Oceanogr.

[16] Southward AJ, Newman WA (1998). Ectosymbiosis between filamentous sulphur bacteria and stalked barnacle (Scalpellomorpha, Neolepadinae) from the Lau Back Arc Basin, Tonga.. Cah Biol Mar.

[17] Miyake H, Kitada M, Tsuchida S, Okuyama Y, Nakamura K (2007). Ecological aspects of hydrothermal vent animals in captivity at atmospheric pressure.. Mar Ecol.

[18] Tsuchida S, Suzuki Y, Fujiwara Y, Kawato M, Uematsu K (2011). Epibiotic association between filamentous bacteria and the vent-associated galatheid crab, *Shinkaia crosnieri* (Decapoda: Anomura).. J Mar Biol Assoc U K.

[19] McCutchan JH, Lewis WM, Kendall C, McGrath CC (2003). Variation in trophic shift for stable isotope ratios of carbon, nitrogen, and sulfur.. Oikos.

[20] Dalsgaard J, John M St, Kattner G, Müller-Navarra D (2003). Fatty acid trophic markers in the pelagic marine environment.. Adv Mar Biol.

[21] Conway N, Kennicutt M, Van Dover C, Lajtha K, Michener R (1994). Stable isotopes in the study of marine chemosynthetic based ecosystems.. Stable isotopes in ecology and environmental sciences.

[22] MacAvoy SE, Macko SA, Joye SB (2002). Fatty acid carbon isotope signatures in chemosynthetic mussels and tube worms from Gulf of Mexico hydrocarbon seep communities.. Chem Geol.

[23] Colaço A, Desbruyeres D, Guezennec J (2007). Polar lipid fatty acids as indicators of trophic associations in a deep-sea vent system community.. Mar Ecol.

[24] DeNiero MJ, Epstien S (1978). Influence of diet on the distribution of carbon isotopes in animals.. Geochim Cosmochim Acta.

[25] Pond DW, Dixon DR, Bell MV, Fallick AE, Sargent JR (1997). Occurrence of 16: 2(n-4) and 18: 2(n-4) fatty acids in the lipids of the hydrothermal vent shrimps *Rimicaris exoculata* and *Alvinocaris markensis*: nutritional and trophic implications.. Mar Ecol Prog Ser 156,.

[26] Paul CK, Hecker B, Comeau R, Freeman-Lynde RP, Neumann C (1984). Biological communities at the Florida Escarpment resemble hydrothermal vent taxa.. Science.

[27] Di Meo CA, Wilbur AE, Holben WE, Feldman RA, Vrijenhoek RC (2000). Genetic variation among endosymbionts of widely distributed vestimentiferan tubeworms.. Appl Environ Microbiol.

[28] Wolff R (2005). Composition and endemism of the deep-sea hydrothermal vent fauna.. Cah Biol Mar.

[29] Vrijenhoek RC, Duhaime M, Jones WJ (2007). Subtype variation among bacterial endosymbionts of tubeworms (Annelida: Siboglinidae) from the Gulf of California.. Biol Bull.

[30] Cavanaugh CM, McKiness ZP, Newton ILG, Stewart FJ (2006). Marine Chemosynthetic symbioses.. Prokaryotes.

[31] Dallwitz MJ, Paine TA, Zurcher EJ (1997). User's guide to the DELTA system.. A general system for processing taxonomic descriptions. 4.08.

[32] Lewis T, Nichols PD, McMeekin TA (2000). Evaluation of extraction methods for recovery of fatty acids from lipid-producing microheterotrophs.. J Microbiol Methods.

[33] Halanych KM, Lutz RA, Vrijenhoek RC (1998). Evolutionary origins and age of vestimentiferan tubeworms.. Cah Biol Mar.

[34] Brodsky LI, Ivanov VV, Kalaidzidis YAL, Leontovitch AM, Nikolaev VK (1995). Genebee-Net – internet-based server for analyzing biopolymers structure.. Biochemistry-Moscow.

[35] Huelsenbeck JP, Ronquist F (2001). MRBAYES: Bayesian inference of phylogenetic trees.. Bioinformatics.

[36] McLaughlin PA, Lemaitre R, Sorhannus U (2007). Hermit crab phylogeny: A reappraisal and its "fall-out.”. J Crust Biol.

[37] Hickerson MJ, Cunningham CW (2000). Dramatic mitochondrial gene rearrangements in the hermit crab Pagurus longicarpus (Crustacea, Anomura).. Mol Biol Evol.

[38] Morrison CL, Harvey AW, Lavery S, Tieu K, Huang Y (2002). Mitochondrial gene rearrangements confirm the parallel evolution of the crab-like form.. Proc R Soc London Ser B.

[39] Yang JS, Yang WJ (2008). The complete mitochondrial genome sequence of the hydrothermal vent galatheid crab *Shinkaia crosnieri* (Crustacea: Decapoda: Anomura): A novel arrangement and incomplete tRNA suite.. BMC Genomics.

[40] Costa FO, deWaard JR, Boutillier J, Ratnasingham S, Dooh R (2007). Biological identification through DNA barcodes: the case of the Crustacea. Can. J. Fish. Aquat. Sci..

[41] Nichols DS (2003). Prokaryotes and the input of polyunsaturated fatty acids to the marine food web.. FEMS Microbiol Lett.

[42] Saito H (2008). Unusual novel n-4 polyunsaturated fatty acids in cold-seep mussels (*Bathymodiolus japonicus* and *Bathymodiolus platifrons*), origination from symbiontic methanotrophic bacteria.. J Chromatrogr A.

[43] Volkman JK, Barrett SM, Blackburn SI, Mansour MP, Sikes EL (1989). Microalgal biomarkers: a review of recent research development.. Org Geochem.

[44] Polz MF, Cavanaugh CM (1995). Dominance of one bacteria phylotype at a Mid-Atlantic Ridge hydrothermal vent site. Proc. Natl. Acad.. Sci USA.

[45] McCaffrey MA, Farrington JW, Repeta DJ (1989). Geochemical implications of the lipid composition of *Thioploca spp*. from the Peru upwelling region – 15^o^S.. Org Geochem.

[46] Zbinden M, Cambon-Bonavita MA (2003). Occurrence of *Deferribacterales* and *Entomoplasmatales* in the deep-sea alvinocarid shrimp *Rimicaris exoculata* gut.. FEMS Microbiol Ecol.

[47] Durand L, Zbinden M, Cueff-Gauchard V, Duperron S, Roussel EG (2010). Microbial diversity associated with the hydrothermal shrimp *Rimicaris exoculata* gut and occurrence of a resident microbial community.. FEMS Microbiol Ecol.

[48] Lesser MP, Weis VM, Patterson MR, Jokiel PL (1994). Effects of morphology and water motion on carbon delivery and productivity in the reef coral, *Pocillopora damicornis* (Linnaeus): Diffusion barriers, inorganic carbon limitation, and biochemical plasticity.. J Exp Mar Biol Ecol.

[49] Jokiel PL (1978). Effects of water motion on reef corals.. J Exp Mar Biol Ecol.

[50] Dennison WC, Barnes DJ (1988). Effect of water motion on coral photosynthesis and calcification.. J Exp Mar Biol Ecol.

[51] Patterson MR, Sebens KP, Olson RR (1991). In situ measurements of flow effects on primary production and dark respiration in reef corals.. Limnol Oceanogr.

[52] Ehrlich H (2010). Biological Materials of Marine Origin: Invertebrates.. Springer.

